# Knowledge, Attitudes, and Practices Regarding Nutrition and Worm Infestation Among Parents/Guardians of Tribal Children of Udupi District: A Cross-Sectional Assessment

**DOI:** 10.1007/s40615-025-02586-4

**Published:** 2025-08-06

**Authors:** Sowmya Pujari, Girish Thunga, Ranjitha S. Shetty, Suneel C. Mundkur, Elsa Sanatombi Devi, Unnikrishnan B., Sreedharan Nair

**Affiliations:** 1 Department of Pharmacy Practice, Manipal College of Pharmaceutical Sciences, Manipal Academy of Higher Education, Manipal, Karnataka 576104 India; 2 Centre for Indigenous Population, Department of Community Medicine, Kasturba Medical College, Manipal, Manipal Academy of Higher Education, Manipal, Karnataka 576104 India; 3 Department of Paediatrics, Kasturba Medical College, Manipal Academy of Higher Education, Manipal, Karnataka 576104 India; 4 Department of Medical Surgical Nursing, Manipal College of Nursing, Manipal Academy of Higher Education, Manipal, Karnataka 576104 India; 5 Department of Community Medicine, Kasturba Medical College, Mangalore, Manipal Academy of Higher Education, Manipal, Karnataka 576104 India; 6 Medical Surgical Nursing Department, Manipal College of Nursing, MANIPAL ACADEMY OF HIGHER EDUCATION, Manipal, Karnataka 576104, India

**Keywords:** Nutritional status, Worm infestation, Koraga tribal children, Knowledge, Attitude, And practices (KAP)

## Abstract

**Background:**

The Koraga tribe, a particularly vulnerable tribal group (PVTG) in India, faces significant health disparities, especially among children. This study focussed on assessing knowledge, attitude, and practices (KAP) regarding nutrition and worm infestation among parents/guardians of Koraga tribal children aged 5 to 10 years in Udupi District.

**Methods:**

A community-based cross-sectional study was conducted among 122 Koraga tribal households to assess the KAP among parents/guardians regarding the nutrition and worm infestation in their children aged 5 to 10 years, using a structured and validated questionnaire developed for this purpose. Descriptive statistics were used to summarize demographic characteristics, and Spearman’s correlation was applied to assess associations between the study variables. Statistical tests were performed using SPSS version 20.0.

**Results:**

Out of the total 122 participants, the majority (94.3%) were females, with a median age of 32 years (IQR 29–36). Most (82%) had a primary level of education, and 56.2% were mothers or female guardians with primary household responsibilities. The median KAP scores were knowledge, 25 (IQR 22–27); attitude, 46 (IQR 44–49), and practice, 23 (IQR 22–25) respectively. Significant positive correlations were found between knowledge and attitude (rho = 0.314, *p* < 0.05) and between knowledge and practice (rho = 0.186, *p* < 0.05). Socio-economic status showed positive correlations with knowledge (*p* < 0.05) and attitude (*p* < 0.05) scores, while the availability of toilet facilities was positively associated with practice scores (*p* < 0.05).

**Conclusion:**

The study revealed that parents/guardians had moderate to good KAP regarding nutrition and worm infestation. The positive correlations suggest an association between higher knowledge, improved attitudes, and better practices, although causality cannot be inferred due to the cross-sectional study design. Future interventional programs may help enhance KAP and promote sustained health benefits among Koraga tribal children.

**Supplementary Information:**

The online version contains supplementary material available at https://doi.org/10.1007/s40615-025-02586-4.

## Introduction

Malnutrition remains a leading cause of child morbidity and mortality in developing countries, particularly affecting children under 5 years of age [1, 2]. Children between 5 and 9 years have increased nutritional demands, and failure to meet these nutritional needs can make children vulnerable to diminished growth, impaired cognitive development, poor academic performance, and decreased productivity [3, 4].

India is known to have a diversified population with approximately 750 indigenous communities known as tribes or *Adivasis*, comprising 8.6% of India’s total population comprises tribal people [5–7]. Among these, the Koraga community, classified as a particularly vulnerable tribal Group (PVTG), resides predominantly in Udupi and Dakshina Kannada districts of Karnataka state and Kasargod district of Kerala. The tribe is internally divided into sub-tribes such as Kuntu, Tappu (Nadsal), Kappada, and Soppu Koragas, each distinguished by differences in language, customs, and rituals. Traditionally residing in structures called *koppa* or *kotta*, the Koragas maintain a lifestyle that reflects a unique blend of tradition and modernity. They practice a matriarchal family system, with a cultural preference for female children. Koragas are traditionally engaged in basket weaving, rope making, and agricultural labour, with some also participating in activities like hunting. They have preserved a wealth of indigenous knowledge, particularly in herbal medicine and natural remedies [8].

The practice of ‘Ajalu’, a dehumanizing ritual involving symbolic subjugation, was officially banned by the Government of Karnataka in 2000 but has left a lasting impact on the community’s social identity [9]. Research by Kamath et al. and Pujar et al. reports widespread anaemia and chronic malnutrition among Koraga women and children, contributing to declining population trends [10, 11]. The health seeking behaviour in this community is often guided by superstitions and prevailing cultural norms [12].

Additionally, soil-transmitted helminth (STH) infections remain a major concern for developing countries, owing mostly to poverty, illiteracy, open defecation, and inadequate personal hygiene. In India, it is found that intestinal parasitic infestations affect approximately 50% of the urban population and 68% of the rural population. Children aged 5 to 10 years are more vulnerable to helminthiasis, which can lead to growth disturbances, cognitive impairment, and increased susceptibility to other infections [13, 14].

KAP assessments related to nutrition are valuable tools for understanding and influencing dietary behaviours and nutritional outcomes. A lack of nutritional knowledge often leads to poor practices, which can result in adverse health consequences. By identifying gaps in knowledge, attitudes and practices, KAP surveys help in developing targeted and effective health interventions [15–17].

Given the scarcity of data on KAP assessments among tribal populations in India, particularly in marginalized communities like the Koragas, this study is important to assess the KAP related to nutrition and worm infestation among parents/guardians of Koraga tribal children aged 5 to 10 years residing in Udupi district. The findings can provide valuable information necessary to develop culturally appropriate, effective interventions to reduce the risk of parasitic infestations and malnutrition in this underserved community. As no standardized KAP tool existed for this population, we developed and validated a context-specific questionnaire as a preliminary step to support this primary assessment.

## Methodology

### Study Design and Setting

A community-based cross-sectional study was conducted among Koraga tribal households residing in rural areas of Udupi District, Karnataka, South India. The study took place across six administrative taluks, namely Udupi, Kaup, Brahmavar, Kundapura, Hebri, and Karkala, from May 2023 to August 2023. The major variables examined in this study were knowledge, attitude, and practice scores related to nutrition and worm infestation, along with relevant socio-demographic factors. The study was reported in accordance with the Strengthening the Reporting of Observational Studies in Epidemiology (STROBE) guidelines [18], with reporting summarized in Supplemental File 1.

### Study Population and Eligibility Criteria

The study included parents/guardians from Koraga tribal households with at least one child aged 5 to 10 years at the time of enrolment. Households were excluded if the child had a birth defect or a serious diagnosed ailment or if the mother was in her third trimester of pregnancy or within 2 to 3 months postpartum.

### Ethics Approval

The study received ethical approval from the Kasturba Medical College and Kasturba Hospital Institutional Ethics Committee, Manipal (IEC-381/2022 [03-04-2023]) before its initiation along with registration on the Clinical Trials Registry—India (CTRI). Written informed consent was obtained from the parents/guardians before their enrolment into the study. Anonymity and confidentiality of the information obtained from the participants were maintained. Additionally, approvals were obtained from the Department of Health and Family Welfare, Government of Karnataka; the Integrated Tribal Development Project, Udupi District; and the District Health Officer, Udupi District.

### Sample Size and Sampling Method

A total of 122 Koraga households with at least one child aged 5 to 10 years were recruited using a criterion-based purposive sampling method considering the limited and geographically scattered nature of the Koraga tribal population [19]. Recruitment was facilitated by Accredited Social Health Activists (ASHAs) and frontline health workers, which ensured effective community outreach, enhanced data quality, and minimized potential for systematic bias.

Purposive sampling across the six taluks of Udupi district allowed inclusion of geographically and culturally diverse households, enhancing the representativeness of the findings. The final sample reflected the maximum feasible recruitment from the eligible tribal population during the study period, ensuring both operational feasibility and adequate geographic representation.

### Study Instrument

A structured KAP questionnaire was developed to assess parental understanding and behaviours related to child nutrition and worm infestation within the Koraga tribal community. Given the absence of a culturally reliable tool for this population, the instrument was developed and validated in several stages [20].

The initial 94-item tool was created through a comprehensive literature review in English and consultations with an expert panel comprising clinicians, academicians, and public health researchers. It was divided into three sections: demographic information (10 items); risk factors (10 items) and KAP-related questions (knowledge, 30; attitude, 14; and practice, 30 items). Knowledge and practice items were scored dichotomously (1 = correct/positive, 0 = incorrect/negative), while attitude items were rated on a 4-point Likert scale, with reverse scoring applied to negatively framed statements.

Content validity was assessed by six professionals from relevant disciplines, including Departments of Pharmacy Practice, Community Medicine, Medical-Surgical Nursing, Paediatrics, and Public Health. The item content validity index (I-CVI) ranged from 0.80 to 1.00 and the scale content validity index S-CVI, computed using the S-CVI/Average (S-CVI/Ave) method, was 0.88, indicating strong content validity [21]. Based on the feedback received, necessary revisions were made to improve clarity and relevance. The finalized questionnaire was then translated into Kannada using standard translation procedures [22, 23].

Face validity was evaluated through pilot testing the Kannada version with 15 Koraga families who met the inclusion criteria. Participants provided feedback on clarity, readability, and overall structure of the questionnaire. Data from the pilot study were not included in the final analysis. The internal consistency of the questionnaire was assessed using Cronbach’s alpha coefficient, which demonstrated acceptable to good internal consistency across domains (knowledge, *α* = 0.750; attitude, α = 0.711; and practice, *α* = 0.867) [24]. The test–retest reliability was assessed by re-administering the tool to the same 15 Koraga families after 1 week. The intra-class correlation coefficient (ICC) for the overall questionnaire was 0.951, indicating excellent reliability [25, 26]. The final validated questionnaire was administered through face-to-face interviews with 122 Koraga tribal families, thereby minimizing the risk of misinterpretation and recall bias.

### Statistical Analysis

All 122 participants completed the full questionnaire with no missing data. No participants were excluded or lost, and all 122 were included in the final data analysis. Therefore, complete case analysis was applied, and no data imputation was required.

Statistical analysis was conducted using IBM SPSS Statistics for Windows, Version 20.0. Armonk, NY: IBM Corp [27]. Participants’ demographic characteristics were summarized using descriptive statistics including median and inter-quartile ranges (IQR) for continuous variables and frequencies (%) for categorical variables.

KAP scores were categorized based on conventions commonly used in KAP literature, including studies by Okello et al. and Wang et al., which adopted Bloom’s cut-off criteria: ≥ 80% as high knowledge, good practice, and positive attitude; 60–79% as moderate knowledge, fair practice, and neutral attitude; and ≤ 59% as low knowledge, poor practice, and negative attitude [28, 29]. The percentage-based cutoffs were calculated based on the proportion of the total obtainable score in each respective KAP domain: 30 marks for each knowledge and practice, and 56 marks for attitude.

The chi-square tests were used to assess the associations between KAP and demographic variables. Spearman’s rank correlation was used to evaluate the relationship between KAP domains, and *p* value < 0.05 was considered statistically significant.

## Results

### Demographic Characteristics

#### Pilot Test

The pilot test involved 15 parents/guardians, with a median respondent age of 35 years (IQR 31–38).

#### Main Study

A total of 122 Koraga tribal families took part in the main study. The median age of the participants was 32 years (IQR 29–36), with a majority being female (94.3%). Socio-economic status was assessed using the modified BG Prasad socio-economic scale, based on the Consumer Price Index (CPI) [30]. Detailed demographic characteristics are presented in Table 1.

**Table 1:** Demographic characteristics of study participants (*N* = 122)

S/N	Demographic variables	Category	Frequency (*n*)	Percentage (%)
1	Gender	Male	7	5.7
		Female	115	94.3
2	Age (years)	18–29	31	25.4
		30–39	82	67.2
		> 40	9	7.4
3	Educational status	No education	5	4.1
		Primary education	100	82
		Higher secondary education	14	11.5
		Graduation	3	2.5
4	Occupation	Homemaker	77	56.2
		Government/private sector	24	17.5
		Others	21	15.3
5	Family type	Nuclear family	78	63.9
		Joint family	44	36.1
6	Number of working members in family	1	53	43
		2	34	27.9
		> 2	35	28.7
7	Socio-economic status^*^	INR > 8397—upper class	92	75.4
		INR 4156–8396—upper middle class	12	9.8
		INR 2460–4155—middle class	12	9.8
		INR 1272–2456—lower middle class	3	2.5
		INR < 1272—lower class	3	2.5
8	Children between 5 and 10 years in family	01	108	88.5
		02	13	10.7
		> 02	1	0.8
9	Eating habits	Vegetarian	**–**	**–**
		Mixed	122	100
10	Toilet facility	Indoor	116	95.1
		No toilet facility	6	4.9

^*^Socio-economic status was classified based on modified BG Prasad socio-economic scale

### Assessment of Knowledge Regarding Nutrition and Worm Infestation

Table 2 summarizes the responses of parents/guardians regarding knowledge related to nutrition and worm infestation of children. The median knowledge score was 25 (IQR 22–27). Most participants had good knowledge (65.6%), followed by fair (26.2%) and poor knowledge (8.2%). Knowledge was particularly strong in areas such as the role of hand hygiene in preventing infections (94.3%), awareness of deworming schedules (100%), and the understanding that walking barefoot increases the risk of worm infestation (95.9%). However, some gaps in knowledge were noted. For instance, only 45.1% correctly identified the appropriate age for introducing complementary food, and 49.2% recognized that clay soil provides a favourable environment for round worms. Additionally, less than 60% were aware that caffeinated beverages are unsuitable for children.

**Table 2:** Responses of the study participants to knowledge-based questions on nutrition and worm infestation (*N* = 122)

S/N	Knowledge statement	Yes	No/don’t know
		*n* (%)	*n* (%)
1	Breastfeeding is very important for child’s immunity	119 (97.5)	3 (2.5)
2	Solid food can be given along with breast milk for babies under 6 months	55 (45.1)	67 (54.9)
3	It is important to give the baby water, honey and other solid food during first 6 months	59 (48.4)	63 (51.6)
4	Green leafy vegetables and fruits can be given to children > 6 months	118 (96.7)	4 (3.3)
5	Breast milk can be supplemented with lactogen in case of less breast milk supply	84 (68.9)	38 (31.1)
6	Meat, fish, eggs and beans are good source of proteins	112 (91.8)	10 (8.2)
7	Low intake of certain fruits and vegetables lead to iron deficiency	92 (75.4)	30 (24.6)
8	Milk is a good source of calcium	94 (77.0)	28 (23.0)
9	Children who do not consume a balanced diet may have scaly skin issues	71 (58.2)	51 (41.8)
10	Loss of appetite is one of the reasons for malnutrition	95 (77.9)	27 (22.1)
11	Caffeine beverages can be given to children can be given to children	72 (59.0)	50 (41.0)
12	Deficiency of vitamin A in children will lead to poor cognitive development	84 (68.9)	38 (31.1)
13	Junk foods and canned drinks can be given to children	96 (78.7)	26 (21.3)
14	Vegetable-based foods should be consumed more frequently than grains and rice-based foods	114 (93.4)	8 (6.6)
15	Cooked food should not be left at room temperature for longer than 2 h	103 (84.4)	19 (15.6)
16	Utensils used to serve food to the children should be washed properly	119 (97.5)	3 (2.5)
17	Unclean hands can transfer germs to the lips, eyes, and nose	115 (94.3)	7 (5.7)
18	Intestines of the human body are the most affected part by worm infestation	101 (82.8)	21 (17.2)
19	Unhygienic conditions are the main cause of worm infestation	106 (86.9)	16 (13.1)
20	Worms enter human body through feco-oral route	95 (77.9)	27 (22.1)
21	Anal itching the common complaint of children with worm infestation	98 (80.3)	24 (19.7)
22	Does stool of the infected child contain worms?	105 (86.1)	17 (13.9)
23	Complications of worm infestation include anaemia, malnutrition, and intestinal obstruction	92 (75.4)	30 (24.6)
24	Worms enter the body through improperly cooked meat	102 (83.6)	20 (16.4)
25	De-worming medicine should be given to children every 6 months	122 (100)	–-
26	Clay soil is the favourable environment for round worms	60 (49.2)	62 (50.8)
27	Diarrhoea and other related diseases can be caused due to unwashed hands	112 (91.8)	10 (8.2)
28	Boiling the drinking water kills the germs	117 (95.9)	5 (4.1)
29	Germs can enter the body by walking bare foot	117 (95.9)	5 (4.1)
30	Worm infestation is one of the main reasons for malnutrition in children	113 (92.6)	9 (7.4)

### Assessment of Attitude Towards Nutrition and Worm Infestation

Parents’ attitudes toward nutrition and worm infestation were assessed through 14 statements, as outlined in Table 3. The median attitude score was 46 (IQR 44–49). Overall, 70.5% of participants had a positive attitude, while 29.5% had a neutral attitude. Most parents expressed strong agreement with healthy behavioural norms, including the belief that children should be taught proper handwashing techniques (92.6%) and that children with worm infestation require medical attention (95.9%). Additionally, 96.7% disagreed with the idea of allowing children to eat in front of the television. However, a small proportion of participants (6.6%) were unsure about the correct timing for introducing complementary feeding, highlighting a need to strengthen attitudes towards early childhood nutrition.

**Table 3:** Responses of the study participants to attitude statements on nutrition and worm infestation (*N* = 122)

S/N	Attitude statement	SA	A	UC	D
I believe that		*n* (%)	*n* (%)	*n* (%)	*n* (%)
1	Instant food is good for children	–-	4 (3.3)	5 (4.1)	113 (92.6)
2	Children should be exposed to variety of foods	25 (20.5)	76 (62.3)	5 (4.1)	16 (13.1)
3	Feeding frequency should be increased according to the child’s age	29 (23.8)	89 (73.0)	1 (0.8)	3 (2.5)
4	Children are healthier if complementary food is introduced at the age of 4 months	11 (9.0)	39 (32.0)	8 (6.6)	64 (52.5)
5	Tea/coffee is good for a child’s health	5 (4.1)	15 (12.3)	8 (6.6)	94 (77.0)
6	It is appropriate for children to have their meals in front of the television	–	2 (1.6)	2 (1.6)	118 (96.7)
7	Combination or a variety of foods if the children refuse to eat	19 (15.6)	77 (63.1)	7 (5.7)	19 (15.6)
8	Children should not be given soft diet (e.g. *ganji-rice porridge*) when they are sick	–-	–-	4 (3.3)	118 (96.7)
9	Education can help in improving the nutritional status	29 (23.8)	79 (64.8)	13 (10.7)	1 (0.8)
10	Children should be advised to wear footwear when they are out playing with soil	34 (27.9)	85 (69.7)	1 (0.8)	2 (1.6)
11	Nails should be kept short and clean to maintain proper hand hygiene	42 (34.4)	79 (64.8)	–-	1 (0.8)
12	Children need not to be taught how to wash hands properly	–-	8 (6.6)	1 (0.8)	113 (92.6)
13	Open defecation is the main reason for worm infestation	29 (23.8)	54 (44.3)	22 (18.0)	17 (13.9)
14	It is not necessary to take the child with worm infestation to the doctor	–-	2 (1.6)	3 (2.5)	117 (95.9)

*SA* strongly agree, *A* agree, *UC* uncertain, *D* disagree

### Assessment of Practice Towards Nutrition and Worm Infestation

Parents/guardians were asked 30 questions to assess their practices for improving nutrition and addressing worm infestation, as shown in Table 4. The median practice score was 23 (IQR 22–25). Among participants, 41% engaged in good practices, 56% in fair practices, and 2.5% in poor practices. Hygiene-related practices were strong, with all families reporting that they washed meat and cleaned kitchen surfaces before cooking. Additionally, 98.4% regularly dewormed their children, and 99.2% reported encouraging their children to wash their hands after handling pets.

**Table 4:** Responses of the study participants to practice-based questions on nutrition and worm infestation (*N* = 122)

S/N	Practice-based questions	Yes	No
		*n* (%)	*n* (%)
1	Does your family consume breakfast every day?	96 (78.7)	26 (21.3)
2	Do you home grow fruits or vegetables?	35 (28.7)	87 (71.3)
3	Do you introduce new foods to children when they refuse to eat?	69 (56.6)	53 (43.4)
4	Do you allow your child to have screen time while having food?	108 (88.5)	14 (11.5)
5	Do you boil the drinking water?	95 (77.9)	27 (22.1)
6	Do you include at least one fruit in your child’s daily diet?	48 (39.3)	74 (60.7)
7	Does your child consume one egg per day?	82 (67.2)	40 (32.8)
8	Does your child consume a glass of milk every day?	48 (39.3)	74 (60.7)
9	Does your child consume minimum of 2 L of water every day?	115 (94.3)	7 (5.7)
10	Do you encourage your child from having sugar-rich foods?	85 (69.7)	37 (30.3)
11	Does your child’s tiffin box contain a balanced diet?	15 (12.3)	107 (87.7)
12	Do you give sufficient time interval (> 3 h) between 2 meals for your child?	120 (98.4)	2 (1.6)
13	Does your child consume packed food items with added additives and preservatives more often?	94 (77.0)	28 (23.0)
14	Does your family consume the cooked meal kept at room temperature within 2 h?	117 (95.9)	5 (4.1)
15	Do you force feed your child if he/she refuses to eat?	61 (50.0)	61 (50.0)
16	Do you consult the paediatrician if child suffers from loss of appetite?	114 (93.4)	8 (6.6)
17	Do you clean the kitchen surfaces before cooking the meals?	122 (100)	–-
18	Do you wash the meat thoroughly before cooking?	122 (100)	–-
19	Does your child wash fruit and vegetables before consumption?	122 (100)	–-
20	Do you encourage your child for open defecation?	110 (90.2)	12 (9.8)
21	Is your child being dewormed every 6 months?	120 (98.4)	2 (1.6)
22	Do you observe the stool of your child after deworming?	52 (42.6)	70 (57.4)
23	Does your family use handwash/soap after using the toilet?	120 (98.4)	2 (1.6)
24	Do you remind your child to wash hands before and after eating?	119 (97.5)	3 (2.5)
25	Do you encourage your children to trim their nails once in a week?	118 (96.7)	4 (3.3)
26	Does your family use in-door latrine facility?	117 (95.9)	5 (4.1)
27	Does your family consume more street food?	99 (81.1)	23 (18.9)
28	Do you opt for home remedies when child complains of abdominal discomfort?	58 (47.5)	64 (52.5)
29	Do you advise your children to wear footwear while playing in soil?	120 (98.4)	2 (1.6)
30	Do you encourage your children to wash their hands after handling their pets?	121 (99.2)	1 (0.8)

### Association of Demographic Features with KAP Scores

The association between demographic characteristics and KAP scores is presented in Tables 5, 6, and 7 (Supplemental File 2, 3, and 4). Among the variables assessed, socio-economic status was the only demographic factor significantly associated with both knowledge and attitude scores (*p* < 0.05). Additionally, the availability of toilet facilities was significantly associated only with practice scores (*p* < 0.05).

**Table 5:** Association of knowledge adequacy with the demographic profile of the study participants (*N* = 122)

Demographic variables	Category	High	Moderate	Low	Chi square*X*^2^	*p*-value
		***n* (%)**	***n* (%)**	***n* (%)**		
Age (years)	18–29	19 (23.8)	9 (28.1)	3 (30.0)	1.070	0.899
	30–39	55 (68.8)	21 (65.6)	7 (70.0)		
	> 40	6 (7.5)	2 (6.3)	0 (0.0)		
Gender	Female	73 (91.3%)	32 (100.0%)	10 (100%)	3.899	0.142
	Male	7 (8.8%)	**-**	**-**		
Educational status	No education	3 (3.8)	2 (6.3)	**-**	3.810	0.702
	Primary education	63 (78.8)	28 (87.5)	9 (90.0)		
	Higher secondary education	11 (13.8)	2 (6.3)	1 (10.0)		
	Graduation	3 (3.8)	**-**	**-**		
Occupation	Homemaker	44 (55.0)	26 (81.3)	7 (70.0)	7.739	0.102
	Government/private sector	20 (25.0)	2 (6.3)	2 (20.0)		
	Others	16 (20.0)	4 (12.5)	1 (10.0)		
Family type	Nuclear family	46 (57.5)	24 (75.0)	8 (80.0)	4.255	0.119
	Joint family	34 (42.5)	8 (25.0)	2 (20.0)		
Number of working members in family	1	29 (36.3)	18 (56.3)	6 (60.0)	8.252	0.083
	2	23 (28.7)	7 (21.9)	4 (40.0)		
	> 2	28 (35.0)	7 (21.9)	**-**		
Socio-economic status	Upper class	61 (76.3)	27 (84.4)	4 (40.0)	23.035	0.003*
	Upper middle class	8 (10.0)	1 (3.1)	3 (30.0)		
	Middle class	9 (11.3)	2 (6.3)	1 (10.0)		
	Lower middle class	2 (2.5)	1 (3.1)	**-**		
	Lower class	**-**	1 (3.1)	2 (20.0)		
Children between 5 and 10 years in family	01	69 (86.3)	29 (90.6)	10 (100)	5.391	0.249
	02	11 (13.8)	2 (6.3)	**-**		
	** > **02	**-**	1 (3.1)	**-**		
Toilet facility	Indoor	77 (96.3)	29 (90.6)	10 (100)	2.110	0.348
	No facility	3 (3.8)	3 (9.4)	**-**		

^*^p < 0.05 indicates statistically significant association (chi-square test)

**Table 6:** Association of attitude adequacy with the demographic profile of the study participants (*N* = 122)

Demographic variable	Category	Positive	Neutral	Chi square*X*^2^	*p*-value
		*n* (%)	*n* (%)		
Age (years)	18–29	21 (24.4)	10 (27.8)	0.205	0.903
	30–39	59 (68.6)	24 (66.7)		
	> 40	6 (7.0)	2 (5.6)		
Gender	Female	80 (93.0)	35 (97.2)	0.827	0.363
	Male	6 (7.0)	1 (2.8)		
Educational status	No education	2 (2.3)	3 (8.3)	3.942	0.268
	Primary education	70 (81.4)	30 (83.3)		
	Higher secondary education	11 (12.8)	3 (8.3)		
	Graduation	3 (5.5)	**–-**		
Occupation	Homemaker	52 (60.5)	25 (69.4)	2.375	0.305
	Government/private sector	20 (23.3)	4 (11.1)		
	Others	14 (16.3)	7 (19.4)		
Family type	Nuclear family	52 (60.5)	26 (72.2)	1.521	0.217
	Joint family	34 (39.5)	10 (27.8)		
Number of working members in family	1	35 (40.7)	18 (50.0)	2.159	0.340
	2	23 (26.7)	11 (30.6)		
	> 2	28 (32.6)	7 (19.4)		
Socio-economic status	Upper class	72 (83.7)	20 (55.6)	12.299	0.015*
	Upper middle class	7 (8.1)	5 (13.9)		
	Middle class	5 (5.8)	7 (19.4)		
	Lower middle class	1 (1.2)	2 (5.6)		
	Lower class	1 (1.2)	2 (5.6)		
Children between 5 and 10 years in family	01	73 (84.9)	35 (97.2)	3.829	0.147
	02	12 (14.0)	1 (2.8)		
	** > **02	1 (1.2)	**–-**		
Toilet facility	Indoor	83 (96.5)	33 (91.7)	1.274	0.259
	No facility	3 (3.5)	3 (8.3)		

^*^p < 0.05 indicates statistically significant association (chi-square test)

**Table 7:** Association of practice adequacy with the demographic profile of the study participants (*N* = 122)

Demographic variable	Category	Good	Fair	Poor	Chi square*X*^2^	Significance
		*n* (%)	*n* (%)	*n* (%)		
Age (years)	18–29	15 (30.0)	16 (23.2)	**-**	4.886	0.299
	30–39	32 (64.0)	49 (71.0)	2 (66.7)		
	> 40	3 (6.0)	4 (5.8)	1 (33.3)		
Gender	Female	49 (98.0)	64 (92.8)	2 (66.7)	5.806	0.055
	Male	1 (2.0)	5 (7.2)	1 (33.3)		
Educational status	No education	1 (2.0)	4 (5.8)	**-**	6.446	0.375
	Primary education	39 (78.0)	58 (84.1)	3 (100)		
	Higher secondary education	7 (14.0)	7 (10.1)	**-**		
	Graduation	3 (6.0)	**-**	**-**		
Occupation	Homemaker	39 (78.0)	36 (52.2)	2 (66.7)	9.303	0.054
	Government/private sector	5 (10.0)	18 (26.1)	1 (33.3)		
	Others	6 (12.0)	15 (21.7)	**-**		
Family type	Nuclear family	35 (70.0)	41 (59.4)	2 (66.7)	1.417	0.492
	Joint family	15 (30.0)	28 (40.6)	1 (33.3)		
Number of working members in family	1	24 (48.0)	28 (40.6)	**1 (33.3)**	0.936	0.919
	2	12 (24.0)	21 (30.4)	1 (33.3)		
	> 2	14 (28.0)	20 (29.0)	1 (33.3)		
Socio-economic status	Upper class	40 (80.0)	50 (72.5)	2 (66.7)	5.185	0.738
	Upper middle class	4 (8.0)	8 (11.6)	**-**		
	Middle class	5 (10.0)	6 (8.7)	1 (33.3)		
	Lower middle class	**-**	3 (4.3)	**-**		
	Lower class	1 (2.0)	2 (2.9)	**-**		
Children between 5 and 10 years in family	01	45 (90.0)	60 (87)	3 (100)	1.241	0.871
	02	5 (10.0)	8 (11.6)	**-**		
	> 02	**-**	1 (1.4)	**-**		
Toilet facility	Indoor	49 (98.0)	65 (94.2)	2 (66.7)	6.205	0.045*
	No facility	1 (2.0)	4 (5.8)	1 (33.3)		

^*^p < 0.05 indicates statistically significant association (chi-square test)

### Correlation Between Knowledge, Attitude, and Practice Variables

Spearman correlation analysis revealed a positive relationship between knowledge and attitude (rho = 0.314, *p* = 0.000) and between knowledge and practice (rho = 0.186, *p* = 0.041). However, there was no statistically significant correlation between attitude and practice (rho =  − 0.014, *p* = 0.875), as depicted in Table 8 (Supplemental File 5). Although statistically significant, the observed correlations were weak, indicating limited associations among knowledge, attitude, and practice scores.

**Table 8:** Correlation among KAP variables of the study participants (*N* = 122)

S/N	Correlation between	Spearman rho	*p*-value	Inference
1	Knowledge-practice	0.186*	0.041	Positive correlation
2	Knowledge-attitude	0.314**	*p* < 0.01	Positive correlation
3	Attitude-practice	− 0.014	0.875	No correlation

^*^Correlation is significant at the 0.05 level (2-tailed)

^**^Correlation is significant at the 0.01 level (2-tailed)

## Discussion

This study offers a valuable insight into KAP regarding nutrition and worm infestation among parents/guardians of the marginalized Koraga tribal community in coastal Karnataka. It utilized a questionnaire that was specifically developed and validated to ensure culturally appropriate and reliable data collection, in alignment with the study’s primary objective.

Overall, most participants displayed adequate knowledge on hygiene and nutrition practices, such as awareness of handwashing and the role of barefoot walking in worm transmission. However, the data also revealed notable knowledge gaps, particularly around complementary feeding, dietary diversity, and the role of micronutrients. These inconsistencies suggest that although basic awareness exists, deeper nutritional literacy is lacking. This highlights the need for more focused and culturally relevant nutritional education. These findings are consistent with the studies by Ene-Obong et al., Siagian et al., Zakaria et al., and Khan et al. which found that good maternal knowledge is positively correlated with improved nutritional outcomes in children [31–34].

Although attitude scores were generally positive, particularly towards discouraging open defecation and feeding junk food to children, some misconceptions persisted, including uncertainty about the timing of introducing solid foods and beliefs surrounding feeding practices. Such attitudes may have been influenced by longstanding cultural norms, limited formal education, and marginalization of the community. Addressing these issues requires culturally sensitive health interventions that align with the lived realities of tribal households.

While hygiene-related practices were strong, such as washing meat before cooking, using soap for handwashing, and regular deworming, dietary practices were suboptimal. Daily fruit and milk consumption, as well as the provision of balanced meals, were reported less frequently in children. This could be attributed to structural challenges, such as poverty, limited access to diverse foods, and the lack of awareness about government nutrition schemes, all of which hinder consistent healthy practices. These findings align with earlier research [35, 36] that links socio-economic status with nutritional behaviour, indicating that interventions must go beyond education to include food security, basic facilities, and community empowerment.

Although weak but statistically significant positive correlations were observed between knowledge and both attitude and practice scores, the cross-sectional nature of the study does not permit causal inference. However, these associations suggest that suitable educational interventions can enhance the knowledge levels, leading to better attitudes and practices related to nutrition and worm infestation.

The strengths of this study include the development and validation of a KAP questionnaire that was tailored specifically to the Koraga community. The study also employed a community-based approach targeting marginalized Koraga tribal families, an area where research is very limited. The questionnaire underwent a rigorous validation process, including content validity assessment, internal consistency, and test–retest reliability testing. These steps ensured that the findings are both credible and relevant.

However, some limitations must be acknowledged. Interpretation of the KAP and health outcomes was challenging owing to the cross-sectional nature of the study. Additionally, the reliance on self-reported data introduces the potential for social desirability bias, which may have affected participants’ responses. Furthermore, the findings may have limited generalizability due to the use of purposive sampling and the focus on a single tribal group with distinct socio-cultural characteristics.

Future longitudinal or interventional studies with repeated follow-ups could provide deeper insights into how KAP evolves over time. Combining direct-observation methods with self-reported data may further enhance data validity. Expanding the research to include other tribal communities may allow for broader comparisons and understanding of community-specific health behaviours. Furthermore, establishing linkages between the KAP outcomes and anthropometric or biomedical indicators in future research could enhance causal interpretations and inform the development of integrated maternal-child health programs for marginalized populations like the Koragas.

## Conclusion

The study’s findings indicate a need for improvement in attitudes and practices regarding appropriate actions to enhance nutritional status and reduce risk for parasitic infestations in young children. Our findings further suggest, but do not establish, that improving knowledge about nutrition and worm infestations among parents/guardians may contribute to the adoption of better practices to improve children’s health. Future research should focus on developing culturally tailored, community-based educational interventions, preferably using longitudinal or interventional study designs to assess changes in KAP over time and establish causal relationships. These research directions align with the Sustainable Development Goals (SDGs) particularly SDG 2 (Zero Hunger), SDG 3 (Good Health and Well-being), SDG 4 (Quality Education), and SDG 6 (Clean Water and Sanitation). Such context-specific approaches are essential to bridging health inequities and promoting sustainable improvements in child health in vulnerable tribal populations.

## Supplementary Information

Below is the link to the electronic supplementary material.

Supplementary file1 (DOCX 34 KB)

## Data Availability

The datasets used and/or analysed during the current study are available from the corresponding author on reasonable request.
